# Special issue: a contemporary landscape of the clinical and biological research in neurofibromatosis type 1

**DOI:** 10.1093/noajnl/vdaa045

**Published:** 2020-06-25

**Authors:** Suganth Suppiah, Sheila Mansouri, Vera Bril, Gelareh Zadeh

**Affiliations:** 1 Division of Neurosurgery, University Health Network, Toronto, Ontario, Canada; 2 MacFeeters-Hamilton Centre for Neuro-oncology, Princess Margaret Cancer Centre, Toronto, Ontario, Canada; 3 Elisabeth Raab Neurofibromatosis Clinic, University Health Network, Toronto, Ontario, Canada; 4 Division of Neurology, University of Health Network, Toronto, Ontario, Canada

Neurofibromatosis type 1 (NF1), first described by Frederich von Recklinghausen in 1882, is one of the most common autosomal-dominant genetic tumor predisposition syndromes.^1^ Individuals with NF1 are born with a germline mutation in the *NF1* gene, which encodes a GTPase activating protein that negatively regulates the RAS signaling cascade. The clinical manifestations of NF1 can vary significantly among affected individuals and thus timely diagnosis can be difficult. NF1 is characterized by the development of numerous benign and malignant tumors that affect multiple organ systems almost anywhere within the body. Common tumors associated with NF1 include neurofibromas, malignant peripheral nerve sheath tumors (MPNSTs), optic pathway gliomas (OPGs), gastrointestinal stromal tumors (GISTs), pheochromocytomas, and leukemias. On account of these malignancies, the life expectancy is shortened in individuals with NF1 to an average of 54 years.^2^ NF1 is an under-recognized and understudied disorder and, as a result, there has been little improvement in the quality of life and overall survival.

The complexity of NF1 and the wide spectrum of clinical complications emphasizes the importance of multidisciplinary teams to provide a holistic approach to patient management. An ideal model of care is for NF1 patients to be managed by a group of multiple specialists with expertise and interest in NF1, including surgeons, oncologists, neurologists, dermatologists, geneticists, genetics counselors, ophthalmologists, psychologists, and others. However, multidisciplinary clinics are rare, particularly for adults with NF1. At present, management paradigms are focused on early detection of treatable complications through routine surveillance and symptomatic treatment. Due to the gaps in our understanding of NF1, the management of patients can vary from institution to institution. There is a tremendous unmet need to establish evidence-based guidelines to build consensus on best practices and ensure dissemination of these approaches in as uniform manner as possible through all centers and physicians treating NF1.

Over the past 3 decades, substantial advances have been made by the neurofibromatosis research community in improving our understanding of this genetic condition. This special issue on neurofibromatosis constitutes a blend of clinical and basic science updates that highlight the research that helped advance the field. The primary aims of this special issue are to increase awareness of the cutting-edge research that is being conducted in the field, examine how this research can be leveraged to improve patient care, and identify gaps in knowledge and care. Here we will highlight a few of the biggest topics in the management of NF1.

## What Are the Drivers of Neurofibroma Development?

- Li et al. “New Insights into the Neurofibroma Tumor Cells of Origin”
- Pundavela et al. “After NF1 Loss in Schwann Cells, Inflammation Drives Neurofibroma Formation”
- Wei et al. “The Impact of the Host Immune Cells on the Development of Neurofibromatosis Type 1: the Abnormal Immune Microenvironment for Tumorigenesis”

*Li et al.* provide a comprehensive historical account of the hunt for the neurofibroma cell of origin. While neurofibromas have been long considered a Schwann cell-derived tumor, the exact initiating cell and the developmental context of NF1 loss of heterozygosity has remained elusive. The 2 major types of neurofibromas, cutaneous and plexiform, have very distinct clinical phenotypes, with plexiform neurofibromas present at birth and developing around nerve plexuses and spinal nerves deep within the body and cutaneous neurofibromas developing at puberty within the dermis. Most notably, only plexiform neurofibromas have a risk of malignant transformation. These differences suggested that cutaneous and plexiform neurofibromas had distinct cells of origin. However, through a series of studies over the past 3 decades, researchers have been able to link the cell of origin of these neurofibroma subtypes to a common stage in Schwann cell development. The leading candidate is the Hoxb7- and Prss56-expressing boundary cap cells/Schwann cell precursor that originates from migrating neural crest stem cells. Plexiform neurofibromas arise from those cells residing in the dorsal root ganglion and spinal nerve roots, while cutaneous neurofibromas arise from dermal glia that migrated from the dorsal nerve roots. Identifying the cell of origin enables accurate modeling of disease initiation and progression, which can help uncover key targetable vulnerabilities within these tumor cells.

After the loss of NF1 in the cell of origin, the immune microenvironment is essential for neurofibroma tumorigenesis. In this issue, *Pundavela et al*. and *Wei et al.* review the role of different immune cells in neurofibroma formation. Schwann cells with loss of NF1 resemble injury-induced repair Schwann cells through Ras-GTP mediated signaling. These cells produce growth factors and cytokines that facilitate the recruitment of immune cells and fibrosis. *Pundavela et al.* propose a model where mast cells and macrophages are recruited to the nerve after the loss of NF1 in Schwann cells. Later, T-cell/dendritic cell recruitment drives neurofibroma initiation and sustains tumor growth. *Wei et al.*, also, examine the role of major histocompatibility complexes and PD-L1 that enable neurofibromas to escape from immune surveillance. The authors suggest that pharmacological modulation of immune recruitment may have therapeutic benefit.

## Development of Targeted Therapies for MPNSTs

- Prudner et al. “Diagnosis and Management of Malignant Peripheral Nerve Sheath Tumors: Current Practice and Future Perspectives”
- Miller et al. “Genetics of Human Malignant Peripheral Nerve Sheath Tumors”
- Terribas et al. “KIF11 and KIF15 Mitotic Kinesins are Potential Therapeutic Vulnerabilities for Malignant Peripheral Nerve Sheath Tumors”
- Godec et al. “Whole Exome Sequencing Reveals the Maintained Polyclonal Nature from Primary to Metastatic Malignant Peripheral Nerve Sheath Tumor in Two Patients with NF1”

MPNSTs are highly aggressive and lethal soft tissue sarcomas that pose a diagnostic and therapeutic challenge, with limited therapeutic options. These tumors represent 15% of all sarcomas, half of which occur in the context of NF1. Despite advances in oncology, the 5-year disease-free survival rate in MPNSTs in NF1 patients remains poor, ranging between 34% and 60%.^3^ Tumor size and tumor grade are prognostic factors for MPNST, which suggest that early diagnosis of this tumor would have benefit. However, there is no consensus on how often NF1 patients should be monitored for MPNST development and what imaging modality would be best. *Prudner et al.* review the current diagnostic and treatment paradigms that have been established for the management of MPNSTs. The gold standard for treatment of MPNSTs is complete surgical resection with negative margins, which has demonstrated significant improvement in survival outcomes.^4,5^ However, the role of neoadjuvant/adjuvant chemotherapy and radiation therapy is an area of debate in MPNST. Large prospective multicenter trials are needed to better elucidate the optimal treatment paradigm, which can prove to be difficult due to the relative rarity of the tumor.

There is a pressing need for the development of targeted therapies to treat MPNSTs. *Miller et al.* provide an in-depth review of the molecular landscape of MPNSTs and lay the foundation to identify potential therapeutic vulnerabilities. It appears that the malignant transformation of neurofibromas into MPNSTs involves sequential loss of tumor suppressors: NF1, CDKN2A/B, and polycomb repressive complex 2 core components. Preclinical studies have demonstrated that MPNST cell lines are sensitive to BRD4-based therapies.^6^ The authors highlight the importance of studies that reveal genes and regulatory pathways that are altered in malignant transformation.

*Terribas et al.* studied the functional role of mitotic kinesins and their potential as a therapeutic vulnerability. Through in vitro studies, the authors demonstrated that kinesins are overexpressed in MPNSTs and required for cell survival. MPNST cell lines were also more sensitive to KIF11 inhibitors (ispinesib and ARRY-520). In addition, co-targeting with KIF11 and BRD4 reduced MPNST cell viability, synergistically killing a much higher proportion of MPNST cells than control fibroblasts. Since single-agent therapies have not shown clinical effect, the results of this study suggest further studies should examine the role of combination therapies targeting multiple oncogenic programs.

Despite wide local surgical resection followed by adjuvant radiotherapy, MPNSTs have a high rate of local recurrence and metastasis. The molecular mechanisms that enable tumor metastasis are not well understood. To address this question, *Godec et al.* investigated the mutational profile of tumors from 2 patients with spatially and temporally distinct metastasis. The authors identified point mutations and copy number losses of *TRIM23* within the metastatic lesions, suggesting that this alteration may be critical for metastatic progression. Furthermore, Trim23 knockdown in MPNST cell lines demonstrated a decreased propensity for metastasis. The results of this study establish TRIM23 as a potential driver of metastasis and a candidate for targeted therapies.

## Development of Targeted Therapies for NF1-Associated Brain Tumors

- Costa and Gutmann “Brain Tumors in Neurofibromatosis Type 1”
- Lobon Iglesias et al. “NF1-like Optic Pathway Gliomas in Children: Clinical and Molecular Characterization of this Specific Presentation”

Individuals with NF1 are at high risk of developing central nervous system tumors, with low-grade gliomas (OPGs and brain stem gliomas) common in early childhood and malignant glioblastomas manifesting in adulthood. *Costa and Gutmann* provide an in-depth review of the clinical and biological basis of gliomas in NF1. Since low-grade gliomas are not routinely biopsied, the authors emphasize the role of the *NF1* murine glioma models in advancing our understanding of the pathogenesis. Use of these *NF1* genetically engineered mouse glioma models was instrumental in discovering the specific progenitor cells that line the third ventricle as the cell-of-origin for OPGs. In addition, these models have permitted interrogation of promising targeted therapies in a preclinical setting prior to translation to human clinical trials. This review highlights the importance of faithful animal models for basic and preclinical translational research. The recently developed genetically engineered NF1 minipig models that better recapitulate the NF1 syndrome may be better suited for further preclinical drug testing.

*Lobon-Iglesias et al.* investigated the possible molecular mechanisms driving OPG in a group of 16 pediatric patients with typical radiological features of NF1-associated OPG but without the NF1 diagnostic criteria. The authors identified RAS–MAPK pathway alterations in 8 of the tumors, including BRAFV600E mutations and BRAF–KIAA oncogenic fusions. In addition, one of these patients had an NF1 nonsense mutation (8% and 70% variant allele frequency in blood and tumor, respectively) suggestive of NF1 mosaicism. Further in vivo and in vitro studies of sporadic and NF1-associated OPGs will help explore the sensitivity to MAPK pathway inhibitors.

## Advancement in the Management of Cutaneous Neurofibromas

- Chamseddin et al. “Management of Cutaneous Neurofibroma: Current Therapy and Future Directions”
- Ortonne et al. “Assessing Interobserver Variability and Accuracy in the Histological Diagnosis and Classification of Cutaneous Neurofibromas”

Cutaneous neurofibromas, a hallmark of NF1, are benign tumors that cause considerable morbidity through physical and psychosocial burden. These tumors are often cosmetically disfiguring and physically distressing due to the hundreds to thousands of lesions present on a single individual.^7^ This often leads to a significant lowering of quality of life through feelings of embarrassment and low self-esteem.^8,9^ There is no one treatment option that is effective for cutaneous neurofibromas and can eradicate these lesions in a ready manner. In this issue, *Chamseddin et al.* review the existing literature on current treatment modalities including surgical excision, CO_2_ laser ablation, photocoagulation, electrodessication, and radiofrequency ablation. Based on the efficacy and limitations of each treatment technique, the authors propose a management algorithm to aid clinicians. Despite the strong evidence that cutaneous neurofibromas cause significant mental health complications, treatment is still often classified as elective and cosmetic by most insurance companies. Further long-term studies are needed to assess the psychosocial and quality-of-life benefits of treating these cutaneous neurofibromas, in order to advocate for increased treatment accessibility. A change in approach to how these lesions are managed requires strong patient-driven advocacy in order to allow the health care team to more effectively address the needs of the patient population.

One of the major challenges with studying cutaneous neurofibromas is the lack of a universally accepted nomenclature to describe the various subtypes. Current terminology varies based on subspecialties and institutions.^10^ For instance, one lesion can be described by multiple terms (nodular, discrete, or localized cutaneous neurofibromas) and, similarly, multiple lesions can be described by a single term (nodular neurofibromas may be intra- or extra-neural). Though seemingly a minor factor, the confusion that stems from this lack of consensus terminology reflects a clear lack of understanding of the etiology and pathophysiology of cutaneous neurofibromas. Advancement of understanding of cutaneous neurofibroma biology is hindered, as we cannot reliably compare the results of different studies. As a first step in developing a unified classification system, *Ortonne et al.* evaluated the interobserver agreement across pathologists in describing and reporting cutaneous neurofibromas. The study demonstrated that there was strong agreement among pathologists that not all neurofibromas involving the skin are cutaneous neurofibromas. However, there was less concordance on classifying cutaneous neurofibroma subtypes based on patterns of growth, composition, extent, and histological subtypes. Identifying the schema and histological features that pathologists use to characterize cutaneous neurofibromas will be the first step toward developing a robust classification system.

## Management of Abdominal Neoplasms in NF1

- Dare et al. “Abdominal Neoplastic Manifestations of Neurofibromatosis Type-1”

A heterogeneous group of abdominal neoplasms are associated with NF1 and pose a major clinical challenge. Up to a quarter of NF1 patients can develop one of these tumors that include plexiform neurofibromas, MPNSTs, rhabdomyosarcomas, pheochromocytomas, and GISTs. These tumors can be difficult to diagnose due to the vague symptoms, multifocal disease, or coexistence of multiple tumor types. In this issue, *Dare et al.* review the literature and present a classification framework for abdominal tumors in NF1. Although minimal evidence exists for the efficacy of screening for abdominal tumors in NF1, a comprehensive screening protocol has improved long-term survival in Li-Fraumeni syndrome, which is another tumor predisposition syndrome. This paper highlights the need for larger multi-institutional studies to study the impact of routine screening for neoplasms in NF1 and its effect on overall survival.

## Design of Clinical Trials to Improve Quality of Life in NF1

- Gross and Widemann “Clinical Trial Design in Neurofibromatosis Type 1 as a Model for Other Tumor Predisposition Syndromes”
- Hamoy-Jimenez et al. “Quality of Life in Patients with Neurofibromatosis Type 1 and 2 in Canada”
- de Blank et al. “Effect of Age and Neurofibromatosis Type 1 Status on White Matter Integrity in the Optic Radiations”

*Gross and Widemann* review the unique challenges to designing clinical trials for the management of nonmalignant manifestations of NF1. Clinical trials for normal malignancies often measure straightforward endpoints, such as overall survival or recurrence-free survival. However, nonmalignant tumors in NF1 are often chronic and not immediately life-threatening, which makes the traditional endpoints of clinical trials in cancer or oncology somewhat irrelevant. These benign tumors may not impact patient life expectancy, but significantly impact the quality of life. Novel therapeutics, therefore, most likely fail based on traditional endpoints, even if they improve the patient’s functional status. The authors highlight the success of the selumitinib clinical trials for benign plexiform neurofibromas in NF1 as a model for the development of trials for other benign tumors. To successfully design similar trials, it is important to properly characterize the natural history of the tumor and identify functional outcome measures that provide a method of assessing the efficacy of new therapeutic agents.

*Hamoy-Jimenez et al.* characterize the quality of life of patients with NF1 and NF2. They utilized patient-completed generic measures such as SF-36, EQ-5D-5L, PROMIS and disease-specific measures such as the PedsQL NF1 module and NFTI-QOL for NF2. The study demonstrated that neurofibromatosis patients suffer a significant reduction in quality of life, as expected. Most notably, the pain was the main driver of physical health and disease visibility was the main driver of mental health. The results of this study highlight potential functional and patient-reported outcome measures that may be used to assess the clinical efficacy of novel therapeutics for nonmalignant tumors.

In addition, the development of noninvasive imaging biomarkers may address the challenges of developing clinical trials in NF1. *de Blank et al.* report their experience with diffusion tensor imaging (DTI) to assess white matter integrity. This study describes DTI measures in 93 children with and without NF1 throughout the brain and specifically the optic radiations. Individuals with NF1 demonstrate increased diffusion throughout the brain. The findings also suggest that children with NF1 may have an altered and delayed maturation of white matter in the developing brain. Better characterization of the trajectory of white matter integrity in children with NF1 may help target early intervention efforts and be used as biomarkers for assessing the efficacy of novel treatments.

## Conclusions

In this special issue, we highlight the current landscape of biological and clinical research in neurofibromatosis. We provide several fundamental recommendations for the future of the field. First, although the last decade has led to significant advancements in our understanding of the biology of tumors in NF1, further developments are necessary and critical for improving treatment paradigms for patients. Identification of molecular alterations driving the malignant transformation of neurofibromas into MPNSTs will be critical to advance development of novel targeted therapeutics and should be done in parallel with the development of reliable preclinical models that allow for rapid translation. In addition to this, standardized core outcomes and definitions that evaluate the quality of life are needed to facilitate the assessment of clinical trials in NF1. Lastly, centers of excellence that are able to provide NF1 patients with multidisciplinary clinics to address the complex needs of these patients in a longitudinal fashion will be key to improving the management of this growing population of patients.
